# Broadband near-infrared metamaterial absorbers utilizing highly lossy metals

**DOI:** 10.1038/srep39445

**Published:** 2016-12-21

**Authors:** Fei Ding, Jin Dai, Yiting Chen, Jianfei Zhu, Yi Jin, Sergey I. Bozhevolnyi

**Affiliations:** 1 Centre for Nano Optics, University of Southern Denmark, Campusvej 55, DK-5230 Odense, Denmark; 2 Department of Materials and Nano Physics, School of Information and Communication Technology, KTH-Royal Institute of Technology, Electrum 229, 16440 Kista, Sweden; 3 Centre for Optical and Electromagnetic Research, State Key Laboratory of Modern Optical Instrumentations, Zhejiang University, Hangzhou 310058, China

## Abstract

Radiation absorbers have increasingly been attracting attention as crucial components for controllable thermal emission, energy harvesting, modulators, etc. However, it is still challenging to realize thin absorbers which can operate over a wide spectrum range. Here, we propose and experimentally demonstrate thin, broadband, polarization-insensitive and omnidirectional absorbers working in the near-infrared range. We choose titanium (Ti) instead of the commonly used gold (Au) to construct nano-disk arrays on the top of a silicon dioxide (SiO_2_) coated Au substrate, with the quality (Q) factor of the localized surface plasmon (LSP) resonance being decreased due to the intrinsic high loss of Ti. The combination of this low-Q LSP resonance and the propagating surface plasmon (PSP) excitation resonance, which occur at different wavelengths, is the fundamental origin of the broadband absorption. The measured (at normal light incidence) absorption is over 90% in the wavelength range from 900 nm to 1825 nm, with high absorption persisting up to the incident angle of ~40°. The demonstrated thin-film absorber configuration is relatively easy to fabricate and can be realized with other properly selected materials.

Thin-film radiation absorbers, which can completely absorb the incident light at specific frequencies within a few hundred nanometers, have drawn increasing attention due to their prospects in many fields of science and technologies, e.g., sensing, thermal emission tailoring and solar energy harvesting^1^,^2^,^3^,^4^,^5^. However, thin-film broadband radiation absorbers are extremely difficult to realize due to natural constraints: very high absorption occurs only near resonances whose bandwidth is necessarily limited, while strong mismatch in dielectric properties with environment (e.g., air) causes high reflection at an interface. Recently, metamaterials have shown their unique capacity in controlling the propagation of electromagnetic waves^6^,^7^,^8^,^9^,^10^. Many thin-film metamaterial absorbers have been demonstrated to function as stable perfect absorbers from the microwave to visible, exhibiting wavelength scalability and insensitivity to the angle of incidence and/or polarization^11^,^12^,^13^,^14^,^15^,^16^. Nevertheless, the aforementioned metamaterial absorbers often tend to be efficient only within a narrow band owing to their resonance property, which might be not sufficient for many practical applications.

To achieve a large absorption bandwidth, a common and direct strategy is to merge several spectrally adjacent resonances by exciting various multi-sized resonators together^17^,^18^,^19^,^20^,^21^,^22^. But the number of the blended resonators is restricted due to the competition of neighboring resonances, and the absorption performance is highly sensitive to the structure size, which is difficult to control in fabrication. Thus the bandwidth broadening is still limited and the experimentally measured absorption efficiency is not very high. In addition to this, there have been other methods addressing this issue, such as adiabatic nanofocusing of gap surface plasmon modes excited by the scattering off subwavelength-sized wedges^23^,^24^ and the excitation of slow-wave modes in tapered alternating metal-dielectric multiple thin films^25^,^26^,^27^,^28^.

Despite these significant achievements, it remains challenging to realize efficient light absorbers with a large bandwidth, high efficiency and fabrication simplicity, since the fabrication of the aforementioned broadband absorbers involved focused ion beam milling (FIB) or complicated multiple steps of electron beam lithography (EBL), which are time-consuming and costly. In this paper, we propose and experimentally demonstrate thin, broadband, polarization-insensitive, and omnidirectional absorbers working in the near-infrared range, which are based on the simple and traditional metal-insulator-metal (MIM) configuration. Highly-efficient broadband absorption is ascribed to the excitation of low-Q localized surface plasmon (LSP) resonance supported by titanium (Ti) nano-disks, and the generation of propagating surface plasmon (PSP) resonance. Specifically, under normal incidence, the measured absorption of the fabricated sample realized by one step of EBL is over 90% in the spectrum ranging from 900 nm to 1825 nm. Moreover, numerical simulations show that the absorption performance is insensitive to the polarization of incident light, and high absorption persists when the incident angle is less than 40°. Such a planar device is much easier to fabricate compared with other broadband absorbers with complicated structures. Additionally, this design concept is general and can be applied to other frequencies with properly selected materials, such as highly-doped semiconductors in THz frequency.

## Results and Discussion

### Structure and design of the broadband absorber

**Figure 1:**
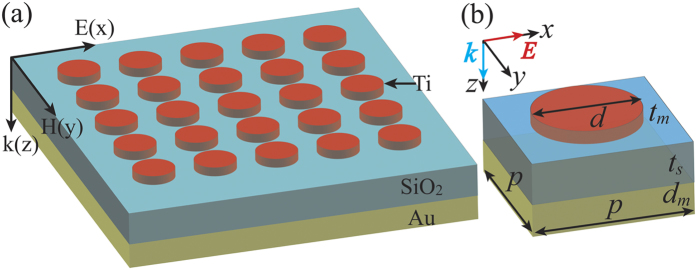
Schematic of the perfect infrared broadband absorber and the incident light polarization configuration. The dimensions are *p* = 600 nm, *d* = 400 nm, *t*_m_ = 30 nm, *t*_s_ = 160 nm and *d*_m_ = 100 nm, respectively.

The schematic of the broadband near-infrared metamaterial absorber is depicted in Fig. 1. The absorber is composed of periodic Ti disk-shape nanoantenna array and a continuous gold (Au) film, separated by a silicon dioxide (SiO_2_) dielectric layer. The topmost disk nanoantennas are periodically distributed in both the *x*- and *y*-directions with a pitch of 600 nm. The other parameters of the disk nanoantenna are *t*_m_ = 30 nm and *d* = 400 nm. The thickness of the middle SiO_2_ spacer layer is optimized to be 160 nm, and the bottom Au layer has a thickness of 100 nm, which is thick enough to block all the transmission in the investigated wavelength range.

### Simulation and experimental results of the broadband Ti-disk absorber

**Figure 2:**
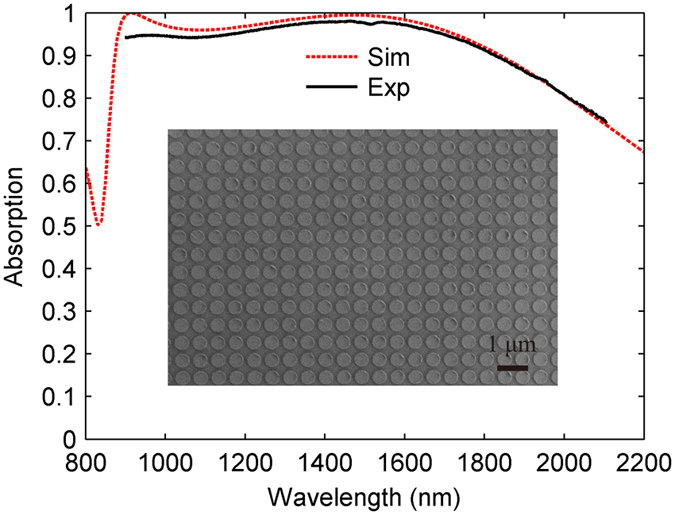
The simulated (red dashed line) and experimental (black solid line) absorption spectra for the fabricated sample. The inset shows the SEM image of a section of the sample.

We first implement three dimensional (3D) full-wave simulations to verify the performance of the broadband absorber. The full-wave simulations are performed with the commercial finite element software Comsol Multiphysics (see the Methods section for details). Figure 1 shows the simulated structure under TM polarization (electric field is along the *x*-direction). The absorption is calculated from the scattering parameters as *A*(*λ*) = 1 − *R*(*λ*) − *T*(*λ*), where *R*(*λ*) represents the reflection, and *T*(*λ*) represents the transmission which is zero here. The obtained absorption spectrum at normal incidence is shown in Fig. 2 (dashed red line), which clearly indicates that above 90% of the incident near-infrared light is efficiently absorbed in a rather wide wavelength range from 875 nm to 1840 nm. The relative bandwidth of the absorber, defined as *RWB* = 2 × (*λ*_l_ − *λ*_s_)/(*λ*_l_ + *λ*_s_), where *λ*_l_ and *λ*_s_ are the long and short limits of a wavelength range with absorption above 90% respectively, reaches about 71%.

To experimentally validate the broadband absorption performance, we fabricated a set of broadband absorbers using the standard thin-film deposition techniques and electron-beam lithography (see the Methods section for details). The inset of Fig. 2 displays a scanning electron microscope (SEM) image of a part of the fabricated sample. All the absorbers cover an area of 75 μm × 75 μm, which consist of 125 × 125 unit cells. Reflection spectra of the fabricated samples are measured using a homemade linear reflection spectroscopy (see the Methods section for details). From Fig. 2, it is impressive that the measured absorption is above 90% in the wavelength range from 900 nm to 1825 nm (black solid curve). The experimentally achieved *RWB* is around 67.9%, which is superior to other reported absorbers based on multi-layered^17^,^24^ or multi-sized^18^,^19^,^20^ structures. Taking into account the imperfect fabrication as well as the measurement error, there is remarkably good agreement between the simulated and experimental results.

**Figure 3:**
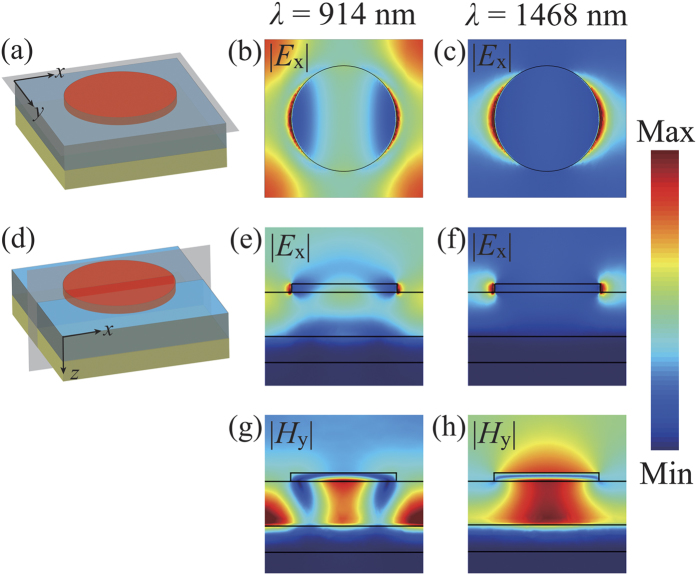
The normalized electric field *E*_x_ and magnetic field *H*_y_ distributions at two absorption peaks in both the *x*-*y* and *x*-*z* planes.

To reveal the physical mechanism in the proposed perfect absorber, we calculate the electric and magnetic field (|*E*_x_| and |*H*_y_|) at the two absorption peaks, namely 914 nm and 1468 nm in Fig. 3. Overall, these two resonances are both the electric dipole resonances excited on the nano-disks along the *x*-axis for TM polarization [Fig. 3(b,c,e,f)]. However, the distributions of the magnetic field in the cross-section [*x-z* plane in Fig. 3(g,h)] are significantly different. The short wavelength resonance at *λ*_1_ = 914 nm is considered to be the PSP resonance between continuous Au film and SiO_2_ spacer, where the magnetic field is not only strongly confined in the gap region underneath the nano-disks, but also strongly enhanced between the nano-disks. Otherwise, the long wavelength mode at *λ*_2_ = 1468 nm is a LSP resonance where the magnetic field is mainly concentrated within the gap between the topmost nano-antennas and the gold underlay^29^. Since Ti is very dispersive and has a relatively large imaginary part (see Supplementary Fig. S1 for details), the intrinsic absorption coefficient of Ti is very big. Thus the Q-factor of this LSP resonance is rather low, broadening the absorption bandwidth, which is different from the narrow band absorber made up of low-damping Au nano-disk array^13^,^14^ (see the following text for details). Therefore, we can attribute the broadband perfect absorption to the combination effect of PSP and low-Q LSP resonances.

**Figure 4:**
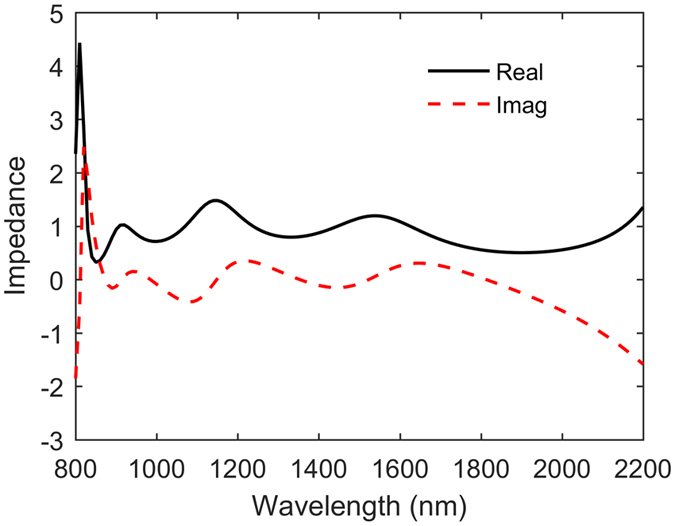
The normalized optical impedance of the Ti-disk broadband absorber at normal incidence.

Since optical impedance matching plays an important role in areas of metamaterial absorbers^30^, we retrieve the corresponding effective optical impedance to get further insight into the physics of our structure. The impedance of our structure can be calculated easily from the scattering parameter results of our numerical simulations, using


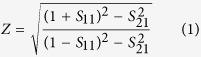


where *S*_11_ and *S*_21_ denote the scattering matrix coefficients of normal incidence reflection and transmission in TM-polarization, respectively. Due to the presence of the thick metallic substrate, *S*_21_ can safely be set to zero. Figure 4 shows the simulation results for the normalized real and imaginary parts of the optical impedance for the Ti-disk broadband absorber. We can clearly observe that both the real and imaginary parts are matched to the respective vacuum values of one and zero in the wide absorption wavelength range, resulting in highly-efficient broadband absorption^30^.

**Figure 5:**
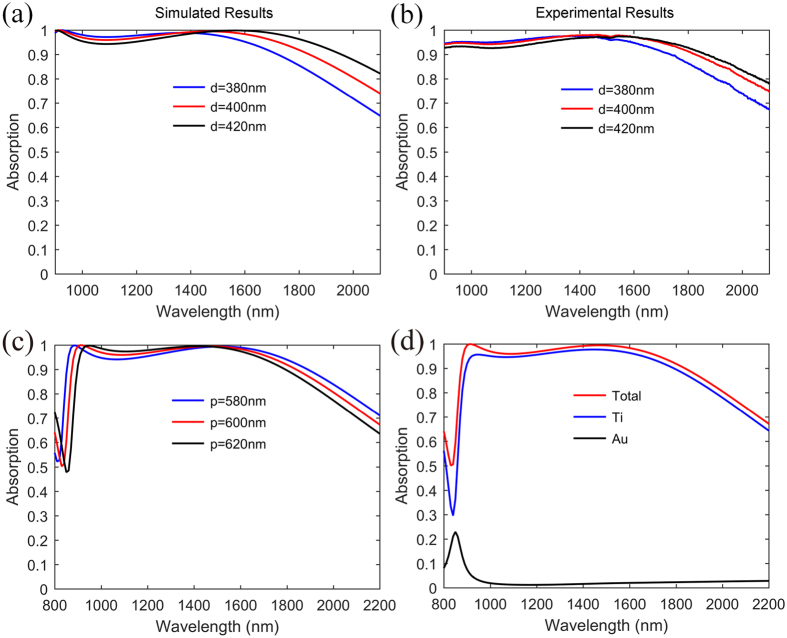
Influence of some structure parameters on the absorption performance and the corresponding absorption spectra of different materials within the absorber. Simulated (**a**) and measured (**b**) absorption spectra when the diameter *d* changes from 380 nm to 420 nm with the other parameter fixed. (**c**) Simulated absorption spectra with different period *p* while the other geometric parameters are fixed. (**d**) Absorption of different materials within this absorber.

After the mechanism of this broadband absorber has been explained, the influence of some structure parameters on the absorption can be easily understood. As discussed above, the absorption peak at *λ*_2_ = 1468 nm is related to the LSP resonance. Thus the diameter *d* of nano-disks may nearly determine the long wavelength limit of *RWB* with the other parameters fixed. Figure 5(a,b) show the corresponding simulated and experimental results with different *d*. As *d* increases from 380 nm to 420 nm, the LSP resonant wavelength is slightly red-shifted, resulting in an increase of *RWB*, which is consistent with previous study. Compared Fig. 5(b) with 5(a), there is remarkable agreement between the simulation and experiment results. However, *d* cannot be too large, otherwise the resonant wavelength difference of PSP and LSP will become too big to overlap and the absorption dip will appear, decreasing the *RWB*.

The absorption spectrum can also be tuned by varying the period *p* of the Ti nano-disk array. Figure 5(c) shows the absorption efficiency as a function of the period *p*. We can observe that as the period *p* increases, the PSP resonance wavelength shifts to the longer wavelength, while the LSP resonance wavelength keeps almost constant. The relation between PSP wavelength and period *p* can be explained by the PSP dispersion relationship, which is given by the following equation,


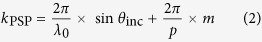


where *k*_PSP_ is the PSP wave vector in the surface of Au-SiO_2_, *λ*_0_ is the wavelength in free space, *θ*_inc_ is the incident angle and *m* is a integer.

To quantify the energy absorbed by different materials within this absorber, we integrated the simple equation of 
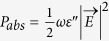
 over some material volume, where *ω* is the angular frequency, *ε*″ is the imaginary part of the permittivity, and 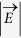 is the amplitude of the electric field. From Fig. 5(d), it is noted that the Ti nano-disks dominate the total absorption in the whole investigated wavelength range, while the absorption of Au layer is weak, which indicates that the Au substrate mainly works as a mirror and contributes little to the direct absorption of the incident light. In the short wavelength range where *λ* < 1000 nm, the Au layer can directly dissipate some of the incident energy, which is attributed to the generated PSP resonance.

### Au-disk based narrowband absorber

**Figure 6:**
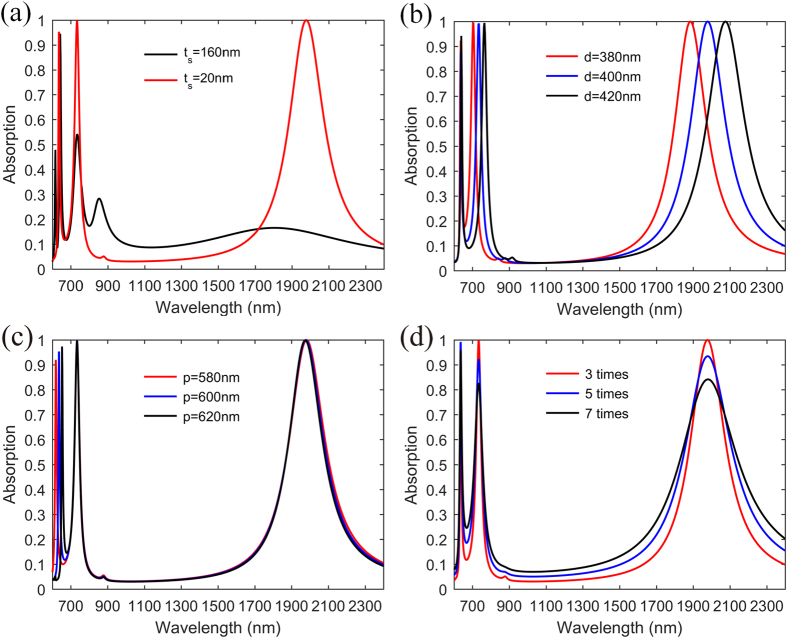
Simulated absorption performance of the Au-disk narrowband absorber. (**a**) Absorption spectra with different dielectric thickness *t*_s_. The other geometric parameters are *p* = 600 nm, *d* = 400 nm, *t*_m_ = 30 nm and *d*_m_ = 100 nm. (**b**) Absorption spectra with different diameter *d* while other geometric parameters are fixed. (**c**) Absorption spectra with different period p while other geometric parameters are fixed. (**d**) Absorption spectra with different damping constants while the geometric parameters are fixed.

**Figure 7:**
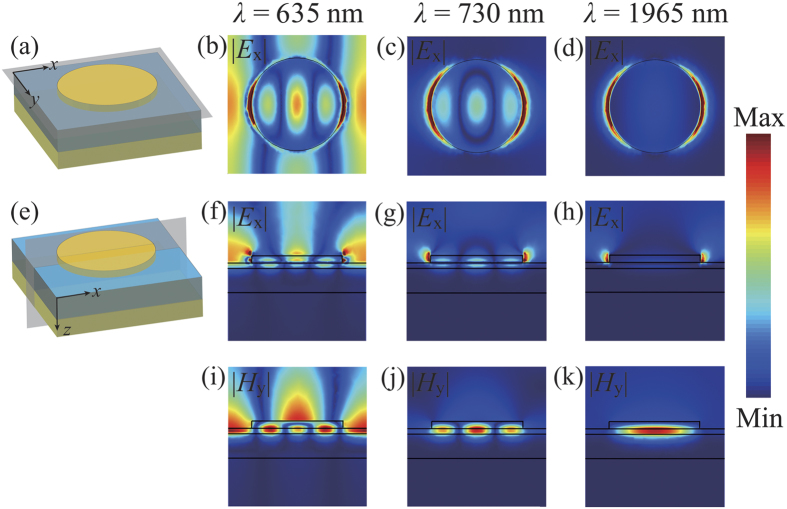
The normalized electric field *E*_x_ and magnetic field *H*_y_ distributions at three absorption peaks in both the *x*-*y* and *x*-*z* planes for the Au-disk absorber.

For comparison, we replace the topmost Ti-disk array with the low loss Au-disk array and achieve a narrowband absorber after numerical optimization [Fig. 6(a)]. To simplify the calculation, here the permittivity of Au is described by the Drude model with the plasma frequency *ω*_*p*_ = 1.37 × 10^16^ s^−1^ and the damping frequency *ω*_*d*_ = 3 × 4.08 × 10^13^ s^−1^, which means the damping constant of the Au film is three times that of bulk Au, owing to the surface scattering and grain boundary effects in thin films^13^. From Fig. 6(a), one can clearly observe three prominent absorption peaks when the thickness of the middle SiO_2_ layer *t*_s_ is reduced to 20 nm. The peak at 1965 nm is a fundamental LSP resonance^13^,^14^ since the magnetic field is confined in the intermediate dielectric layer effectively and there is no coupling between adjacent disks [Fig. 7(k)]. At short wavelength around 730 nm, higher order LSP resonance is excited [Fig. 7(c,g,j)], resulting in highly-efficient narrowband absorption. This resonance exhibits higher Q factor compared to the fundamental LSP resonance as the fundamental LSP resonance has higher radiative loss. Additionally, these two LSP resonances show rapid red-shifting with regard to increasing disk diameter *d*, and are nearly insensitive to the period *p* [Fig. 6(b,c)]. One may notice that there is a sharp absorption peak at 635 nm, which is red-shifting quickly if the period *p* is increased. From the electric and magnetic fields [Fig. 7(b,f,i)], we can see that this peak corresponds to a hybrid mode which involves PSP between continuous Au film and SiO_2_ spacer and high-order LSP resonance. In addition, the PSP resonance is dominating as the field is mostly extended along the surface-parallel direction and the absorption peak is almost independent of the disk diameter *d*. Though there is slight interaction with high-order LSP resonance, this absorption peak can match well with the calculated PSP wavelength (see Fig. S2 for details).

If the damping constant of Au film is increased, the absorption peaks with different amplitudes are observable [Fig. 6(d)]. Furthermore, the full width at half maximum (FWHM) of the absorption peaks are getting broader, decreasing the Q factor. One may imagine that if the damping constant is further increased, lower Q resonances can be excited. Based on the low-Q resonances, broadband highly-efficient absorption can be expected after structure optimization.

### Incident-angle-insensitive absorption performance

**Figure 8:**
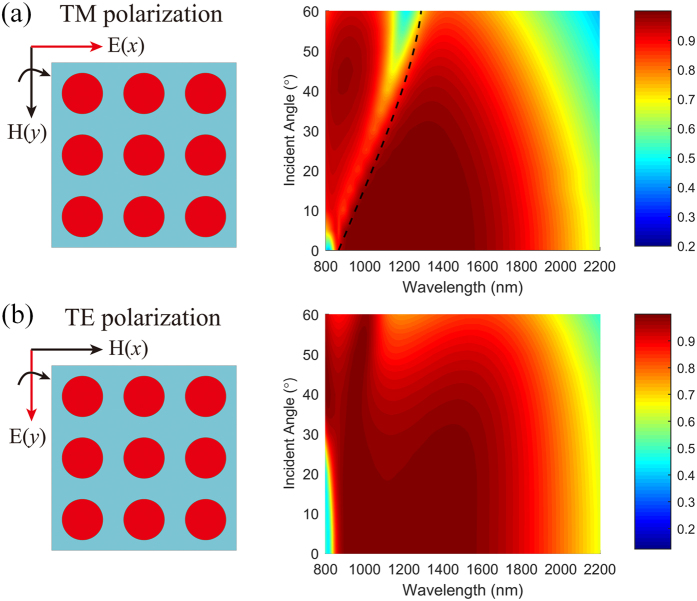
Dependence of the absorption performance on the incident angle for TM (**a**) and TE (**b**) polarizations. The black dashed curve is the calculated dispersion relation of PSP.

In practical applications, such as photovoltaic solar-thermal power generation, the absorption should be as robust as possible for non-normal incidence. To verify the angle dependence of the absorber, we performed full-wave simulations for both TM polarization (the magnetic field of the incident light is kept parallel to the *y*-axis) and TE polarization (the electric field of the incident light is kept parallel to the *y*-axis), as shown in Fig. 8. In the simulation, the incident angle (*θ*) is varied from 0° to 60° in steps of 5°. From Fig. 8(a) one can clearly see an obvious red-shift of the PSP resonance and there appears an absorption dip in the investigated wavelength range as the incident angle increases, resulting in lower absorption efficiency and narrower bandwidth. The black dashed line shows the dispersion relation of PSP calculated using Eq. (1), which matches the simulation results very well. Though this PSP resonance does cause a slight discontinuity on the absorption spectra, the overall absorption performance for TM polarization is still good. For TE polarization, the absorption effect is nearly robust for a relatively wide range of incident angles. From Fig. 8(b), it can be seen that the ultra-broadband absorption response can be achieved when *θ* is below 40°, and the absorption still remains above 80% even when *θ* reaches 60°.

## Conclusion

In conclusion, we have proposed a type of thin near-infrared broadband, omnidirectional and polarization-independent absorbers based on the simple MIM configuration. Due to the high optical loss of Ti, absorption exceeding 90% is experimentally observed over a wide spectrum ranging from 900 nm to 1825 nm by combining the low-Q LSP resonance and the generated PSP resonance on Au-SiO_2_ interface. It should be emphasized that our approach can naturally be exploited with other metals with high loss, for example, nickel (Ni) and tungsten (W). The simulated absorption of Ni based absorber is shown in Fig. S3. In addition, this method can be implemented with highly-doped semiconductors in the THz regime^31^,^32^. Such a feature may find numerous applications in controllable thermal radiation and energy harvesting.

## Methods

### Simulations

The full-wave simulations are performed using the commercial finite element software Comsol Multiphysics. In the simulations, we only model a single unit cell by applying periodic boundary conditions on the vertical sides of the cell. The incident wave is assumed to be a plane wave propagating normal to the surface with the polarization being either *x*-polarized (TM) or *y*-polarized (TE). In our Au-SiO_2_-Ti configuration, the permittivities of Au and Ti are described by interpolated experimental values^33^,^34^, while SiO_2_ is considered to be lossless with a constant refractive index of 1.45. In the Au-SiO_2_- Au configuration, the permittivity of Au is described by Drude model for simplicity. The medium above the Ti nano-disks is chosen to be air. The air domain is truncated using a port boundary that is transparent for the reflected light, while a perfect electric conductor boundary condition is applied on the bottom side of the optically thick Au substrate.

### Fabrication

The absorbers are fabricated on a silicon substrate onto which successive layers of 3 nm Ti, 100 nm Au, 3 nm Ti and 160 nm SiO_2_ are deposited using electron-beam evaporation (metals) and RF-sputtering (SiO_2_). The film growth rates for Ti, Au and SiO_2_ are 0.05 nm/s, 0.1 nm/s, and 0.05 nm/s, respectively. The absorber is patterned using electron-beam lithography (30 kV acceleration voltage) on a 100 nm thick PMMA resist film (Micro Chem 950 K A2). After exposure, the sample is developed in a solution of methyl isobutyl ketone (MIBK) and isopropyl alcohol (IPA) of MIBK: IPA = 1:3 for 35 s. Once the development of the resist is complete, a 30 nm Ti layer is deposited sequentially using thermal deposition (0.02 nm/s). After a lift-off process performed using acetone, the Ti patterns are finally formed on top of the SiO_2_ film.

### Measurement

Reflection spectra of the fabricated samples are measured using a homemade linear reflection spectroscopy, which includes a microscope (Olympus) and a fiber-coupled spectrometer (Ocean Optics NIR-QUEST). The reflected light is collected in the backscattering configuration using an MPlanFL (Olympus) objective with a magnification of ×50 (numerical aperture (N.A.) = 0.75) and normalized to the corresponding intensity of the beam reflected from a gold film (200 nm thick).

## Additional Information

**How to cite this article**: Ding, F. *et al*. Broadband near-infrared metamaterial absorbers utilizing highly lossy metals. *Sci. Rep.* **6**, 39445; doi: 10.1038/srep39445 (2016).

**Publisher's note:** Springer Nature remains neutral with regard to jurisdictional claims in published maps and institutional affiliations.

## Supplementary Material

Supplementary Information
