# Supplementary material for: Broadband near-infrared metamaterial absorbers utilizing highly lossy metals
Source: Sci Rep. 2016 Dec 21;6:39445. doi: 10.1038/srep39445 (PMC5175172; doi:10.1038/srep39445)
Supplement: Supplementary Information [file srep39445-s1.pdf]

Supplementary information for

Broadband near-infrared metamaterial absorbers utilizing highly lossy metals

Fei Ding,[1,*] Jin Dai,[2] Yiting Chen,[1] Jianfei Zhu,[3] Yi Jin,[3] and Sergey I. Bozhevolnyi[1]

[1]*Centre for Nano Optics, University of Southern Denmark, Campusvej 55, DK-5230 Odense, Denmark*

[2] *Optics and Photonics, School of Information and Communication Technology Royal Institute of Technology (KTH), Kista 16440, Sweden*

[3] *Centre for Optical and Electromagnetic Research, State Key Laboratory of Modern Optical Instrumentations, Zhejiang University, Hangzhou 310058, China*

[*]*feid@iti.sdu.dk*

Relative Permittivity of Ti

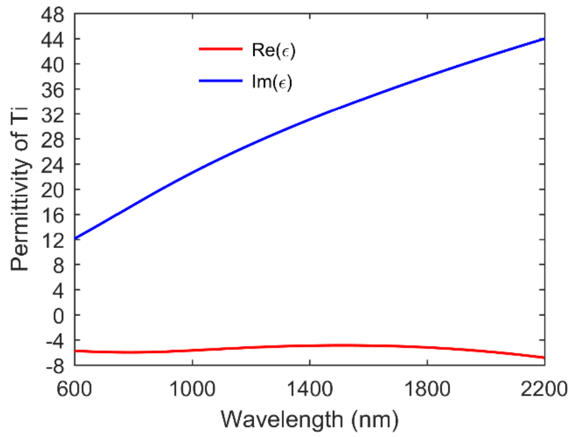

Supplementary Figure S1. Relative permittivity of Ti.

Calculation of the PSP Wavelength

In the calculation, we simply take the SiO_2-Au configuration into consideration. The calculated PSP wavelength for different incident wavelengths is shown in Fig. S2. It should be noted that though the presence of the top Ti (or Au) nanodisks slightly perturbs the PSP's field distribution and mode index from its idea case, i.e. without the nanodisks, it can still reasonably exhibit the PSP properties. If the thickness of SiO_2 t_s is 160 nm, the calculated PSP wavelength of 600 nm corresponds to the incident wavelength at 929 nm, which is in reasonable agreement with the simulated peak at 914 nm considering the approximation of the calculation model. Meanwhile, once t_s is reduced to 20 nm, the mode index of PSP resonance decreases, approaching 1. To excite the PSP resonance at 600 nm in the Au-SiO_2-Au configuration, the incident wavelength equals to 636 nm, matching perfectly with the simulated PSP absorption peak at 635 nm. With the periodicity of 600 nm in our study, the corresponding PSP resonances of the two different configurations can be effectively excited.

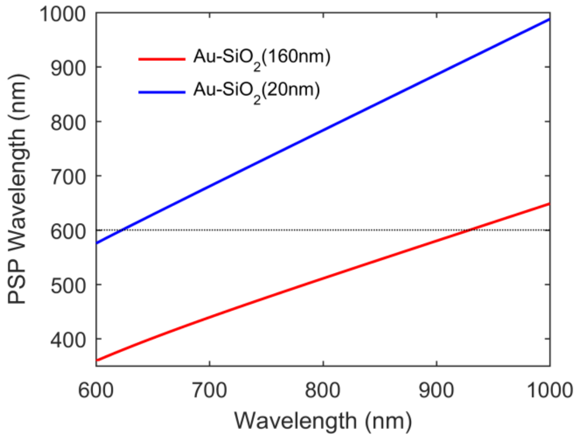

Supplementary Figure S2. The calculated PSP wavelength of the SiO_2-Au configuration.

Ni-disk Array Based Broadband Near-infrared Absorber

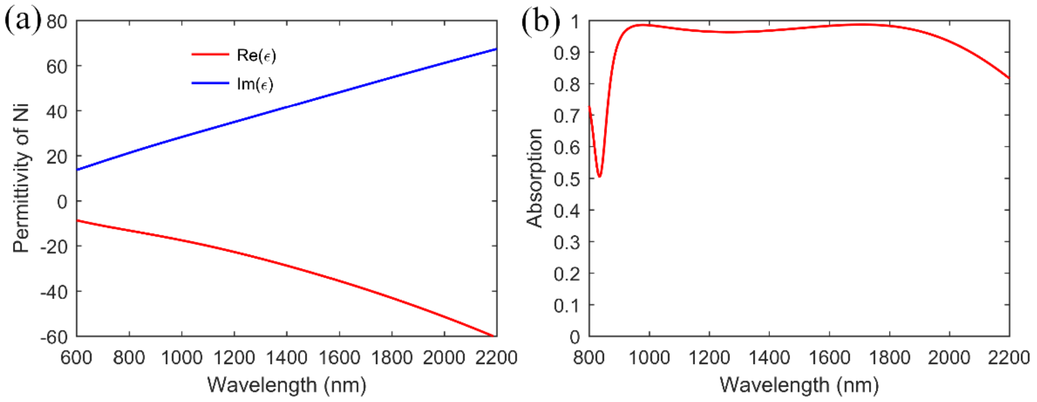

Supplementary Figure S3. Relative permittivity of Ni (a) and the simulated absorption of the Ni-disk array absorber (b). The absorber is composed of the Ni nano-disk array, middle SiO_2 layer and the Au substrate, and the configuration is the same as that shown in Fig. 1. The dimensions are p = 600 nm, d = 400 nm, t_m = 15 nm, t_s = 160 nm and d_m = 100 nm, respectively.
